# The duality between particle methods and artificial neural networks

**DOI:** 10.1038/s41598-020-73329-0

**Published:** 2020-10-01

**Authors:** A. Alexiadis, M. J. H. Simmons, K. Stamatopoulos, H. K. Batchelor, I. Moulitsas

**Affiliations:** 1 School of Chemical Engineering, University of Birmingham, Edgbaston, Birmingham, B15 2TT UK; 2 College of Medical and Dental Sciences, University of Birmingham, Edgbaston, Birmingham, B15 2TT UK; 3 Strathclyde Institute of Pharmacy and Biomedical Sciences, University of Strathclyde, 161 Cathedral Street, Glasgow, G4 0RE UK; 4 Centre for Computational Engineering Sciences, Cranfield University, Bedford, MK43 0AL UK

**Keywords:** Computational models, Machine learning

## Abstract

The algorithm behind particle methods is extremely versatile and used in a variety of applications that range from molecular dynamics to astrophysics. For continuum mechanics applications, the concept of ‘particle’ can be generalized to include discrete portions of solid and liquid matter. This study shows that it is possible to further extend the concept of ‘particle’ to include artificial neurons used in Artificial Intelligence. This produces a new class of computational methods based on ‘particle-neuron duals’ that combines the ability of computational particles to model physical systems and the ability of artificial neurons to learn from data. The method is validated with a multiphysics model of the intestine that autonomously learns how to coordinate its contractions to propel the luminal content forward (peristalsis). Training is achieved with Deep Reinforcement Learning. The particle-neuron duality has the advantage of extending particle methods to systems where the underlying physics is only partially known, but we have observations that allow us to empirically describe the missing features in terms of reward function. During the simulation, the model evolves autonomously adapting its response to the available observations, while remaining consistent with the known physics of the system.

## Introduction

**Figure 1:**
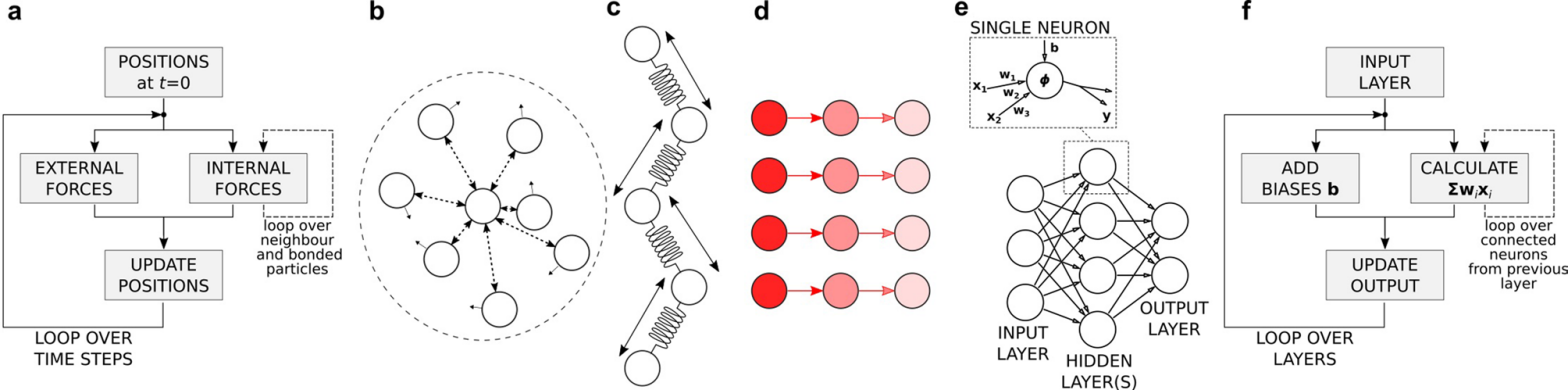
Functioning of particle methods and link with Artificial Neural Networks. (**a**) Typical flow of particle methods algorithms. (**b**) Particle methods like molecular dynamics, smoothed particle hydrodynamics or the discrete element method exchange forces among non-bonded particles. (**c**) Methods like the lattice spring model or peridynamics exchange forces among bonded particles. (**d**) In discrete multiphysics, heat transfer occurs by exchanging heat among neighbouring particles. (**e**) Artificial neural networks exchange information among interconnected neurons. (**f**) The algorithm for forward propagation in ANNs has the same flow as the particle methods algorithm.

The family of particle-methods comprise of a variety of computational techniques that range from molecular dynamics to computational astrophysics. Despite the different fields of application, however, almost all particle methods follow the same algorithm (Fig. 1a). Firstly, the computational domain is subdivided in discrete entities (i.e. computational particles) that, according to the circumstances, represent atoms, portions of matter, solid objects, or even entire stars. Secondly, the forces exchanged among the particles are calculated. Finally, based on these forces, the particles’ positions are updated by solving Newton’s equation of motion. The difference between one particle method and another lies essentially on how these forces are calculated and what type of physical phenomena are represented. For instance, in Molecular Dynamics (MD), they represent the attractive and repulsive forces between atoms^1,2^; in the Lattice Spring Model (LSM) or in Peridynamics (PD), the elastic forces occurring in solids^3,4^; in Smooth Particle Hydrodynamics (SPH), the pressure and viscous forces occurring in fluids^5,6^; and, in direct gravitational N-body simulations, gravity between stars^7^. Because of their common algorithm, particle-methods can also be coupled together in Discrete Multiphysics (DMP) simulations^8–11^. Fluid structure interactions, for instance, combine solid and liquid particles: the LSM calculates the forces occurring in the solid, SPH the forces in the fluid; and additional forces (e.g. repulsive forces to prevent compenetration between phases) model the solid–liquid interface. Moreover, DMP simulations can include heat or mass transfer^12,13^: the algorithm in Fig. 1a still applies, but updates properties other than position, and calculates physical interactions other than forces. In the case of heat transfer, for instance, the new property is temperature, and, besides forces, particles also exchange heat.

What makes particle methods so versatile is their inherently simple concept: discrete objects interact with each other and, by exchanging some type of information (e.g. forces or heat), update their properties (e.g. position or temperature). Given its fundamental nature, it should not come as a surprise that, sometimes in disguise, this concept is often found in other algorithms. This study shows that Artificial Neural Networks (ANNs) are one of those algorithms and it is possible to unify ANNs and particle methods under the same framework. Within this framework, it is possible to define a particle-neuron dual that combines the ability of computational particles to correctly reproduce the physics with the ability of artificial neurons to autonomously learn how to improve the (physical) model during the simulation.

A previous study of ours^14^ coupled DMP with Reinforcement Learning. That article did not make use of ANNs. However, in the conclusions, it mentioned the particle-neuron duality as a theoretical possibility that could arise if Reinforcement Learning were replaced by Deep Reinforcement Learning. DMP was linked with ANNs in another study^15^, where the duality was introduced in the context of a classification problem requiring Supervised Learning (i.e. cell-sorting in a microfluidic device). To the best of our knowledge this is the first time that the particle-neuron duality is established in the context of Deep Reinforcement Learning.

## The particle-neuron duality

As mentioned, particle methods function by exchanging forces among neighbouring particles. This can occur in different ways. Non-bonded particles (e.g. fluid particles) move during the simulation, and the neighbours, with which they exchange forces, are not always the same (Fig. 1b). On the contrary, bonded particles (e.g. solid particles) only exchange forces with predetermined particles (Fig. 1c). Additionally, particles can have different types of interactions; in heat transfer problems, for instance, they exchange heat instead forces (Fig. 1d).

We can look at ANNs from a similar angle. Similarly, to computational particles, artificial neurons are discrete computational elements: they do not exchange forces or heat, but general (i.e. not necessarily physical related) information (Fig. 1e). They do not have position or temperature that varies under the effect of forces or heat, but more general outputs that varies under the effect of inputs. The algorithm for forward propagation in ANNs (Fig. 1f) is also surprisingly similar to the particle methods algorithm (Fig. 1a). We can take advantage of these similarities and design a mathematical object that is, at the same time, a computational particle and an artificial neuron. When these objects exchange forces (or heat), they behave like computational particles; when they exchange non-physical related information, they behave like artificial neurons. We call these new mathematical objects *particle-neuron duals* and a mathematical model based on particle-neuron duals a *Deep Multiphysics* (DeepMP) model. In the next section, we discuss how this can be implemented in practice.

## Discussion and conclusions

**Figure 2:**
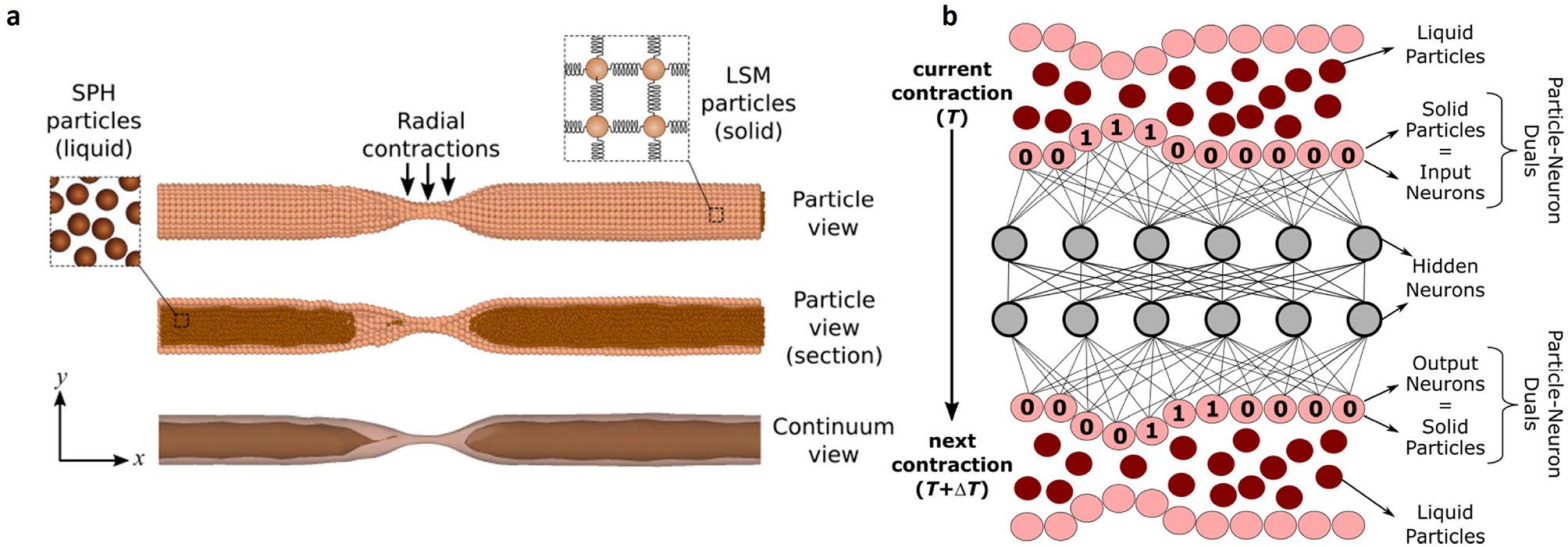
The DeepMP model combines a DMP model with an ANN. (**a**) DMP model: SPH particles model the fluid, LSM particles model the membrane. (**b**) Liquid particles are computational particles that only exchange forces; hidden neurons are computational neurons that only exchange (non-physical) information; solid particles are particle-neuron duals that exchange forces with the other computational particles, and information with the hidden neurons. Given the state of the membrane at time *T*, the ANN calculates which section of the membrane (and for how long) should be contracted next to maximize the amount of fluid moved from left to right.

Consider a fluid in a flexible pipe. The pipe can contract any of its parts squeezing the fluid completely or partially out of the contracted section (Fig. 2a). We can loosely think of this system as a section of the intestine and at the liquid as the luminal content. The objective of the DeepMP model is to learn, without being explicitly programmed to do so, how to simulate the process of peristalsis by coordinating its contractions and move the luminal fluid from left to right. Peristalsis is only one of the many motility patterns occurring in the intestine; the goal of the model is not to reproduce the complexity of the real intestine, but only peristaltic coordination. We choose peristalsis because this type of motility has the specific purpose of effectively propelling the contents distally. Thus, the expected outcome of the training is somehow predictable providing a visual baseline for evaluating the performance of the model.

The DeepMP model combines a DMP model that calculates the physics, and an ANN that learns peristalsis (Fig. 2b). The DMP model accounts for two types of particles, SPH particles that model the fluid and LSM particles that model the solid: details of the DMP model are given in the Methods section. The DMP model can contract specific sections, but it is not specifically programmed to move the fluid in any given direction. Random contractions would simply move the fluid back and forth; and only a well-coordinated sequence will result in the overall motion of the fluid’s centre of mass.

In the intestine, coordination is controlled by the Enteric Nervous System (ENS), but the DMP model alone cannot replicate the effect of the ENS. To achieve this goal in our simulations, we provide the model with an ‘artificial brain’ (Fig. 2b) that, based on the current status of the pipe, determines which section should be contracted next (and for how long). This artificial brain is a fully connected ANN with four layers that takes as input the particles contracting at time *T* (current state of the membrane) and output the particles that will contract at time *T* + Δ*T* (next action based on the current state of the membrane). In Fig. 2b, the membrane particles subjected to contraction are ‘activated’ and given a value of 1; the non-contracting particles are ‘inactive’ and given a value of 0. This information is gathered into a vector of ones and zeros that represents the input layer of the ANN. The input layer passes the information to two hidden layers with 50 neurons each. After they process the information, the ANN outputs a vector of the same size of the input: each element of the vector corresponds to a particle of the membrane. If the value of the vector is 1, the membrane is contracted at that location. The ANN updates its state and decides a new course of action every Δ*T* = 1 s. It is also possible it continues squeezing the same section for longer; in this case, the input and the output vectors are the same. To take advantage the axial symmetry, we group membrane particles in concentric ‘slices’ (see “Methods” section).

The SPH particles that model the fluid are pure computational particles, since they only exchange forces (with each other and with LSM particles). The LSM particles that model the membrane are particle-neuron duals. They exchange forces (with each other and with SPH particles), but, since they also constitute the input and output layers of the ANN, they also exchange information with the hidden layers. The hidden neurons are pure computational neurons, since they only exchange information (with each other and with LSM particles). All particles, neurons and particle-neuron duals belong to the same computational domain, but the neurons are ‘invisible’ because do not have a position property that is updated by forces.

At this stage, the ANN does not yet perform correctly as it needs to be trained and learn an effective strategy for coordinating the contractions. This is different from traditional ANNs that are trained by adjusting the weights to maximize the ability of the network to classify a series of observations. In this case, the ANN must learn a policy that produces the best strategy to achieve a given goal. In order to train the network, we use Deep Reinforcement Learning (DRL) and, specifically, Q-learning (QL), which is the algorithm behind AlphaGo, the computer program that defeated the human word champion at the board game Go^16^. The reader can refer to Neftci and Averbeck^17^ and Lapan^18^ for details on DRL and QL. In the Methods section, we discuss how DRL is applied to our specific case.

**Figure 3:**
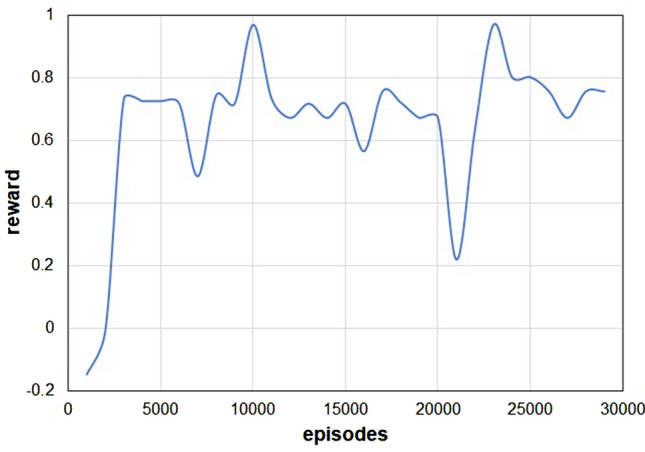
Training of the DeepMP model. Evolution of dimensionless cumulative reward during training showing catastrophic forgetting.

The DRL algorithm is designed to maximize the ‘reward’ during the training of the ANN. In our case, we define the reward as the variation of the centre of mass of the fluid (more details in the “Methods” section). Figure 3 shows how the dimensionless cumulative reward changes during the training of the network. The dimensionless reward is defined as the ratio between the total change of volume of the contracting sections and the volume of liquid displaced forward. If the value of the dimensionless reward is close to one, the algorithm is very efficient in pushing the luminal content forward. If it is zero, the liquid is equally split in the two directions. If it is negative, the liquid overall flows backwards.

Initially, the ANN is not trained; hence this produces random, non-coordinated, contraction (see Video 1). After 2,000 episodes, the model develops an effective, but not yet optimal, strategy (see Video 2). Finally, after 10,000 episodes the model fully masters the task at hand (see Video 3). The dimensionless reward never reaches unity since, as Video 3 shows, a small amount of the liquid leaks back during the episode. The amplitude of the contraction is such that the tube closes almost completely, but, as it also occurs in human peristalsis^19^, this does not prevent that, from time to time, a small amount of liquid leaks back during the contraction. After training, the propulsion mode of the model is similar to that observed in the intestine of humans and experimental animals^19^. Moreover, in the human intestine^19^, the peristaltic wave travels at speeds between 0.1 and 1 cm s^−1^; in the model, the speed is around 0.6 cm s^−1^. Another feature of Fig. 3 is the periodic drop of performance affecting the learning phase. This is a relatively common phenomenon in DRL known as *catastrophic forgetting*. There are various techniques to mitigate it, but they are not employed here since the performance after 10,000 episodes is considered satisfactory.

This example shows how DeepMP provides a new paradigm for coupling physics-based models with Artificial Intelligence (AI). Commonly, if we know the physics behind a given phenomenon, we model it based on first-principles (FPs); if we do not know the physics, but we have (big) data, we use Machine Learning (ML). In practice, most engineering problems are in between: we know some of the physics, but not everything; we have some data, but not enough for ML. DeepMP is designed for this type of ‘middle-ground’ problems. It does not require perfect knowledge of the FPs because the AI component will improve the physical model during the simulation. It does not require big data, because the physical model will generate new data during the simulation. DeepMP only requires observations that help identifying the nature of the reward in the DRL algorithm. In this study, for instance, we can model the mechanics of both the membrane and the luminal content by FPs, but we cannot model the complexity of the Enteric Nervous System by FPs. DeepMP bypasses this issue. We know the goal of peristalsis is to efficiently move the luminal content forwards; this observation is enough to identify a reward for the DRL algorithm (i.e. variation of the centre of mass of the fluid). Once the reward is set, the ANN learns peristalsis based on the data generated by the FP model during the simulation.

Because of its features, DeepMP differs from any other technique that couples FP modelling with ML. These techniques can be divided in two groups. In the first group, the FP model generates data that are fed into the ML algorithm, e.g. for dimensionality-reduction^20,21^. In the second group, the ML algorithm generates a data-driven model that is fed into the FP model^22,23^. These approaches have their benefits, but, because they link the FP model and the ML algorithm in series, they still require either a complete knowledge of the FPs or a large quantity of data. If the first element of the series is the FP model, complete knowledge of the physics is required; if it is the ML algorithm, big data are required. This is not the case of DeepMP that is designed for scenarios where we have partial knowledge of the FPs and limited amount of data.

This study also anticipates a question that is likely to become increasingly important in the next years: i.e. what is the most effective way to couple Multiphysics models with Reinforcement Learning?

DRL is rapidly becoming the new frontier of ML. However, the physical models currently used by the Reinforcement Learning community to simulate the interaction between the agent and the physical environment are very simple, almost trivial, if compared with the tools developed in the last twenty years by the Multiphysics community. Often, physical models with less than 100 degrees of freedom are considered ‘complex’ and ‘high-dimensional’ in DRL^24^. The DMP model used in this study, which is small for multiphysics standards, accounts for 14,578 three-dimensional particles, which implies 43,734 degrees of freedom. So far, this question has remained somehow in the background because both Reinforcement Learning and Multiphysics are computationally intensive, and it is currently impractical to couple multiphysics models of millions of degrees of freedom with extensive DRL based on ANNs with millions of artificial neurons. However, things are changing rapidly: computational power is increasing at great strides and quantum computers are around the corner. Therefore, it is not too soon to ask the question on how complex multiphysics models and Reinforcement Learning should be best coupled together; Deep Multiphysics, based on the underlying duality between computational particles and artificial neurons, provides a first answer to this question.

## Methods

### Discrete multiphysics model

The length of the tube in Fig. 2a is 6 × 10^−1^ m, the diameter 5 × 10^–3^ m, the density of the fluid 1000 kg m^−3^, and the viscosity 10^−1^ Pa s. The fluid is divided in 12,078 computational particles with mass 2.5 × 10^–4^ kg and modelled with SPH^25^. Pressure forces are calculated with the Tait equation

1 $$ P = \frac{{c_{0} \rho_{0} }}{7}\left[ {\left( {\frac{\rho }{{\rho_{0} }}} \right)^{7} - 1} \right] $$

where *c*_0_ and *ρ*_0_ are reference velocities and densities. Viscous forces between particles *i* and *j* are calculated with

2 $$ {\Pi }_{ij} = - ah\frac{{c_{0} }}{{\rho_{ij} }}\frac{{v_{ij} r_{ij} }}{{r_{ij}^{2} + bh^{2} }} $$

where *h* = 9 × 10^–3^ m is the smoothing length, *v*_*ij*_ the relative velocity between the two particles, and *ρ*_*ij*_ = *ρ*_*j*_ + *ρ*_*i*_. The parameter *a* depends on the kinematic viscosity^25^, while *b* = 0.01 ensures the stability of the simulation. Periodic boundary conditions are used, i.e. particles that exit the computational domain from one side of the tube, re-enter from the other side.

The flexible pipe is divided 2500 particles with mass 2.5 × 10^–4^ kg modelled with the LSM^26^. Elastic forces between two particles *i* and *j* are calculated using Hooke’s law

3 $$ F_{ij} = k(r_{ij} - r_{0} ) $$

where *k* = 9 × 10^–2^ N m^−1^ is the Hookean constant, *r*_0_ = 6 × 10^–3^ m the equilibrium distance between the particles, and *r*_*ij*_ their instantaneous distance. An additional Hookean spring with *k′* = 5 × 10^–4^ N m^−1^ is used to tether each particle of the membrane to its initial position and return the particles to their initial position after the contraction. Contractions are achieved by adding an axisymmetric force *f*_0_ = 4 × 10^–4^ N pointing towards the axis of the pipe to all particles of a given section. This section can be located everywhere along the tube, but its thickness is always one tenth of the pipe length. Fluid–solid interaction is achieved with a repulsive potential of the type

4 $$ U_{ij} = A\left[ {1 + \cos \left( {\frac{{\pi r_{ij} }}{{r_{0} }}} \right)} \right], \, r < r_{0} $$

with *A* = 2 × 10^–6^ J, to avoid compenetration, while no-slip boundary conditions are approximated by exchange viscous forces like those of Eq. (2) between liquid and solid particles. The time step used in the simulations is ∆*t* = 10^–3^ s. The DMP model was derived from a previous study^27^, where more details and additional numerical considerations (e.g. convergence of the results with respect to the size and the number of particles used in the model) can be found.

### Deep reinforcement learning (DRL)

When the tube contracts at a certain location, we perform an ‘action’ *a*. As a result of this action, the flexible membrane changes shape and, therefore, its ‘state’ *s*. If we contract the tube on a different location, we perform a new action *a′* and the system acquires a new state *s′*. In our case, every state is a 10-dimensional vector of zeros and ones that constitutes the input of the ANN (i.e. which particles are contracting at time *T*). Actions are also represented by 10-dimensional vectors of zeros and ones that constitute the output of the ANN (i.e. which particles are going to be contracting at time *T* + *∆T*). Every action produces a movement *∆x* of the centre of mass of the fluid. If the change of centre of mass of the fluid is in the positive axial direction, *∆x* is positive and we call it the ‘reward’ *r* of the action *a*. If the change is in the opposite direction, *∆x* is negative and produces a negative reward. During a simulation, we perform *N* actions *a* that bring to *N* corresponding states *s* and *N* rewards *r*: the series of *N* sets (*a*, *s*, *r*) is called an ‘episode’. The objective of DRL is to train the ANN in such a way that given an initial state, the ANN determines a series of actions that maximize the cumulative reward of the whole episode. To achieve this goal, RL introduces the concept of ‘quality’ *Q*(*s*, *a*) of an action *a* given the state *s*

5 $$ Q(s,a) = r + \gamma \mathop {\max }\limits_{{a^{\prime}}} Q(s^{\prime},a^{\prime}) $$

where max *Q*(*s′*, *a′*) returns the maximum *Q* value for the best possible action *a’* in the next state *s′*, and the parameter *γ* (< 1) is the *discounting factor* that weights the importance of future rewards on *Q*. In DRL, an ANN is used to approximate *Q*(*s*, *a*); the multiphysics model will then execute the action with the highest quality, i.e. max *Q*(*s*, *a*). To achieve this goal, Eq. (5) must be the training target of the ANN, which implies a loss function of the type

6 $$ L(s,a) = \left[ {\underbrace {{r + \gamma \mathop {\max }\limits_{{a^{\prime}}} Q^{*} (s^{\prime},a^{\prime})}}_{{{\text{target}}}} - \underbrace {{Q^{*} (s,a)}}_{{\text{ANN output}}}} \right]^{2} $$

where Q^*^(*s*, *a*) is the ANN approximation of *Q*(*s*, *a*). At the beginning of the training, the values of Q^*^(*s*, *a*) are meaningless because the ANN is initialized with random weights. If the action is selected only based on max Q^*^(*s*, *a*), the model would always repeat the same actions: it will learn from these actions, but they will be far from optimal. Therefore, we need a way to force the model to explore alternatives to max Q^*^(*s*, *a*). To achieve this, from time to time, actions are selected randomly. The probability α to choose a random action over max Q^*^(*s*, *a*) is higher at the beginning of the training and decays as it progresses. In our case, initially α = 1 (fully random) and decays linearly to α = 0.1 during 30,000 episodes.

The simulations of the physical model are carried out with the open-source software LAMMPS^28^ compiled as a Python library, while the Python library Keras is used for training the network. Details are given in Table 1: the reader can refer to the Keras documentation^29^ for the items in Table 1 not explicitly discussed in the text. In Table 1, the size of the output layer is 10. However, in the most general case, the size of the output layer should be 2^10^. In fact, if *a* is a 10-dimensional vector (of ones and zeros indicating which sections are contracting or relaxing at time *T* + ∆*T*), the number of all possible *a* (e.g. all possible contraction patterns) is *N*_*a*_ = 2^10^. The ANN, therefore, takes *s* (size 10) as input and gives Q^*^(*s*, *a*) (size 2^10^) as output. However, if we assume that only one section at the time can be contracted, the size of the output layer reduces to 10, which is consistent with the description of the ANN given in Fig. 2b.

**Table 1:** Architecture of the ANN and Hhyperparameters used for training.

Input layer	N = 10	
Hidden layer 1	N = 50	*ϕ* = relu
Hidden layer 2	N = 50	*ϕ* = relu
Output layer	N = 10	*ϕ* = linear
Hyperparameters	Loss = mse	Optimizer = adam
	Metrics = mae	*γ* = 0.5
	α = 1.0–0.1	Episodes = 30,000

The optimal model has a dimensionless reward close to 1 (which is close to theoretically maximal efficiency). The model was also trained with a settings (e.g. architecture of the ANN, hyperparameters) different from those of Table 1, and some of the runs produced suboptimal models, which are not shown here. Visualization (Fig. 2a) and Videos were processed with the software Ovito^30^.

## Supplementary information

Supplementary Information.

Supplementary Video Legends.

Supplementary Video 1.

Supplementary Video 2.

Supplementary Video 3.

## Data Availability

The code used for the simulations is freely available under the GNU General Public License v3 and can be downloaded from the Cranfield repository https://public.cranfield.ac.uk/e102081/DeepMP/.
