# Publisher Correction: The duality between particle methods and artificial neural networks

**DOI:** 10.1038/s41598-021-85463-4

**Published:** 2021-03-05

**Authors:** A. Alexiadis, M. J. H. Simmons, K. Stamatopoulos, H. K. Batchelor, I. Moulitsas

**Affiliations:** 1 School of Chemical Engineering, University of Birmingham, Edgbaston, Birmingham, B15 2TT UK; 2 College of Medical and Dental Sciences, University of Birmingham, Edgbaston, Birmingham, B15 2TT UK; 3 Strathclyde Institute of Pharmacy and Biomedical Sciences, University of Strathclyde, 161 Cathedral Street, Glasgow, G4 0RE UK; 4 Centre for Computational Engineering Sciences, Cranfield University, Bedford, MK43 0AL UK

Correction to: *Scientific Reports* 10.1038/s41598-020-73329-0, published online 01 October 2020

This Article contains a typographical error in the Code availability section.

“The code used for the simulations is freely available under the GNU General Public License v3 and can be downloaded from the Cranfield repository https://public.cranfield.ac.uk/e102081/DeepMP/.”

should read:

“The code used for the simulations is freely available under the GNU General Public License v3 and can be downloaded from the Cranfield repository http://public.cranfield.ac.uk/e102081/DeepMP/.”

