# Supplementary material for: The duality between particle methods and artificial neural networks
Source: Sci Rep. 2020 Oct 1;10:16247. doi: 10.1038/s41598-020-73329-0 (PMC7530753; doi:10.1038/s41598-020-73329-0)
Supplement: Supplementary file 1 — Supplementary Information. [file 41598_2020_73329_MOESM1_ESM.docx]

**The duality between particle methods and artificial neural networks**

Alessio Alexiadis^a*^, Mark J. H. Simmons^a^, Konstantinos Stamatopoulos^a*^, Hannah K. Batchelor^b,c^, Irene Moulitsas^d^

^a^ School of Chemical Engineering, University of Birmingham, Birmingham, Edgbaston, Birmingham B15 2TT, United Kingdom

^b^ College of Medical and Dental Sciences, University of Birmingham, Birmingham, Edgbaston, Birmingham B15 2TT, United Kingdom

^c^ Strathclyde Institute of Pharmacy and Biomedical Sciences, University of Strathclyde, 161 Cathedral Street, Glasgow G4 0RE, United Kingdom

^d^ Centre for Computational Engineering Sciences, Cranfield University, Bedford MK43 0AL, United Kingdom

^*^Corresponding author, email: a.alexiadis@bham.ac.uk

^**^Corresponding author, email: k.stamatopoulos@bham.ac.uk
