# Supplementary material for: The duality between particle methods and artificial neural networks
Source: Sci Rep. 2020 Oct 1;10:16247. doi: 10.1038/s41598-020-73329-0 (PMC7530753; doi:10.1038/s41598-020-73329-0)
Supplement: Supplementary file 2 — Supplementary Video Legends. [file 41598_2020_73329_MOESM2_ESM.docx]

**Legend for the videos:**

Video 1: Non coordinated contraction at the beginning of the training phase.

Video 2: Model performance after 2,000 training episodes.

Video 1: After 10,000 training episodes, the model learns peristalsis.
